# Systematical Ingredient Investigations of *Ficus tikoua* Bur. Fruit and Immunoregulatory and Antioxidant Effects of Different Fractions

**DOI:** 10.3390/molecules27206880

**Published:** 2022-10-14

**Authors:** Yu Gong, Wei Luo, Hulan Chen, Bo Ren, Weicheng Hu, Limei Li

**Affiliations:** 1 College of Pharmacy, Southwest Minzu University, Chengdu 610041, China; 2 Key Laboratory of Standardization of Chinese Herbal Medicine, Ministry of Education, State Key Laboratory of Southwestern Chinese Medicine Resources, Chengdu University of Traditional Chinese Medicine, Chengdu 611137, China; 3 Jiangsu Collaborative Innovation Center of Regional Modern Agriculture & Environmental Protection, Jiangsu Key Laboratory for Eco-Agricultural Biotechnology around Hongze Lake, Huaiyin Normal University, Huaian 223300, China

**Keywords:** *Ficus tikoua* Bur., nutrient, polysaccharide, antioxidant, immunomodulation

## Abstract

Although the fruit of *Ficus tikoua* Bur. has been consumed by montanic people in China for centuries, its chemical and biological composition was still unclear. A series of comprehensive investigations on its chemical constituents and bioactivities were carried out for the first time. As a result, six compounds were isolated and identified as the main components in this fruit. GC–MS analysis of the lipid components demonstrated that *Ficus tikoua* Bur. fruit contains some wholesome constituents such as fatty acids, vitamins, triterpenoids, and phytosterols. The fatty acids are mainly composed of linolenic acid (61.27%) and linoleic acid (22.79%). Furthermore, this fruit contains a relative high content of crude protein (9.41 ± 0.03%), total amino acids (9.28%), and total polyphenols (0.86 ± 0.01 g/100 g). The analysis of monosaccharide composition showed that the total polysaccharide mainly consists of glucose, glucuronic acid, xylose, arabinose, mannose, galactose, galacturonic acid, and rhamnose. The polysaccharide, polyphenol, water, ethanol, and flavonoid extracts exhibited prominent antioxidant activity determined by ABTS, DPPH, and FRAPS methods. Meanwhile, the total polysaccharide exhibited significant immunomodulatory effect by enhancing the release of cytokines and expression of iNOS and COX-2 in RAW264.7 cells, significantly decreasing the expression of c-Jun and p65 proteins in the cytoplasm; increasing the translocation of c-Jun and p65 to the nucleus; and regulating the phosphorylation level of Akt, PI3K, and PDK1 in the PI3K/AKT signaling pathway. This study proved that the fruit of *F. tikoua* is a reliable source of functional food.

## 1. Introduction

*Ficus tikoua* Bur. is a kind of prostrate vine growing on wastelands, grassy banks, sandy hillsides, and open woodland, within an altitude of 500–2000 m. It belongs to the genus *Ficus* of the Moraceae family and is widely distributed throughout south China, northeastern India, Laos, and Vietnam [1]. As a traditional folk medicine, the stems and leaves have been used to treat rheumatic pain, acute gastroenteritis, dysentery, and other diseases [2]. Chemical constituent and bioactivity investigation showed that the stems and leaves mainly contain antifungal isoflavonoids [3], antioxidant lignans and phenolic compounds [4,5], flavanones as α-glucosidase inhibitors [6], and coumarins [5]. Furthermore, isoflavonoids with antioxidant and alpha-glucosidase inhibitory activities were found from the rhizomes of *F. tikoua* [7]. Isoflavanones were obtained from the whole plant of *F. tikoua,* and some of them exhibited cytotoxicity [8]. The ripe fruit of *F. tikoua* turns dark red and has a diameter of 1–2 cm, spherical or oval, with multiple round tumor spot structures on surface (Figure S1 Supplementary Material). It is also called ‘wild melon’ in China because its ripe fruit looks like ruby and smells pleasant, which is so highly appreciated by rural kids and residents as alternative sources of fruit that some local people have begun to culture it. Many studies showed that wild fruits usually foster complex phytochemical compositions and accumulate active compounds like polyphenols. Some wild berries are rich in nutrients and exhibit diverse bioactivities such as antidiabetic, antioxidant, and anti-proliferative activities [9,10]. Nowadays, wild fruits are attracting more attention because of their rich nutrients and active ingredients, such as protein, vitamins, amino acids, polyphenols, and flavonoids, which are beneficial to human beings, showing a tremendous potency in the functional food market [11].

Although *F. tikoua* fruit has been consumed as a wild fruit in mountain areas for centuries and its volatile components were preliminarily analyzed by GC–MS [12], its other phytochemical composition and nutrient contents, as well as biological capacity, were poorly understood. In this study, its chemical constituents and contents of phytochemicals, together with bioactivities including antioxidant capacity by FRAP, ABTS, and DPPH assays and in vitro immunological activity, were systematically investigated, aiming to provide a comprehensive understanding of this fruit for consumers and lay a scientific foundation for the development and exploitation of *F. tikoua* fruit.

## 2. Results and Discussion

### 2.1. Chemical Constituents

**Figure 1:**
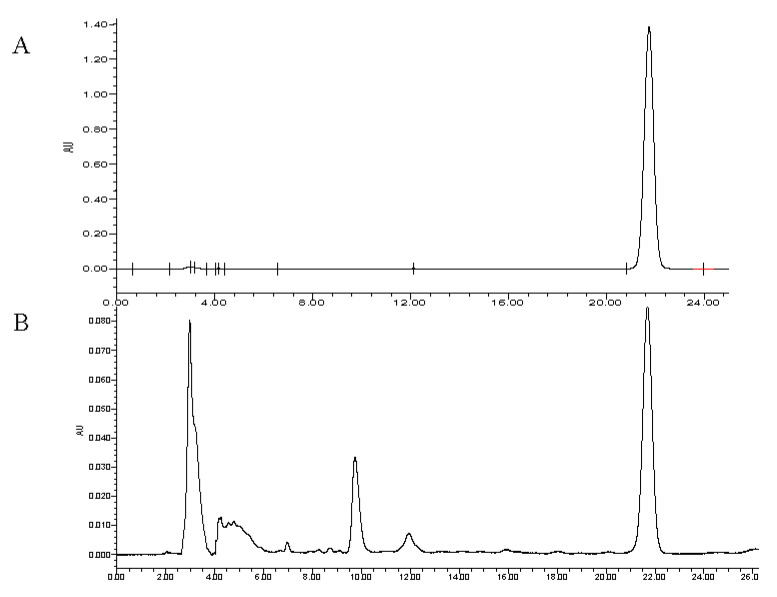
(**A**) HPLC chromatogram of psoralen standard. (**B**) HPLC chromatogram of psoralen in *F. tikoua* fruit.

Six known compounds were isolated from the fruit of *F. tikoua*, and they were identified as *β*-sitosterol (**1**) [13], stigmasterol (**2**) [14], psoralen (**3**) [15], 5-methoxypsoralen (**4**) [16], protocatechuic acid methyl ester (**5**) [17], and daucosterin (**6**) [13]. What is more, it is pretty noteworthy that there was always a pleasant scent during extraction and isolation, and psoralen and its analogues were eventually found to contribute to its aroma. Psoralen has been a well-known medicine in photochemotherapy for skin diseases such as vitiligo since 1947 [18]. On the other hand, previous studies showed that the combination of furocoumarins and UV irradiation is carcinogenic, and the intake of food rich in psoralen and furocoumarins may be related to a higher incidence of melanoma [19]. As shown in Figure 1A,B, the fruit contains a relatively high content of psoralen (876.9 mg/kg), which is comparable with those plants rich in coumarins, e.g., *Citrus aurantifolia* Swingle (334 mg/kg) and *C. latifolia* Tanaka (502 mg/kg) [20]. In view of the high level psoralen, it has potential to be a dietary supplement for the treatment of skin diseases. However, in case of skin cancer, people should be wary of long-time sunshine when eating a large amount of this fruit. Protocatechuic acid methyl ester is a natural antioxidant, widely present in fruits. As a main constituent of this fruit, the content of protocatechuic acid methyl ester was quantitatively determined to be 460.0 mg/kg by HPLC.

In order to fully understand the chemical constituents in this fruit, the total methanol extract was first analyzed by LC–MS (Figures S2 and S3), which is an efficient way to identify known compounds in a complex system by measuring accurate *m*/*z* signals and fragments of each peak. In total, 28 compounds were identified (Table S1), including 6 amino acids (asparagine, *D*-(+)-proline, pipecolic acid, *DL*-norleucine, *L*-phenylalanine, and betaine), 4 alkaloids (choline, 8-hydroxyquinoline, 4-indolecarbaldehyde, and indole-3-acrylic acid), 6 organic acids (*D*-(−)-quinic acid, *DL*-malic acid, citric acid, methylmalonic acid, neochlorogenic acid, and chlorogenic acid), 2 disaccharides (*D*-(+)-maltose and sucrose), a flavone (8-prenylnaringenin), a coumarin (psoralen), and 8 fatty acid derivatives (9*S*,13*R*-12-oxophytodienoic acid, 9-oxo-10(*E*), 12(*E*)-octadecadienoic acid, *α*-eleostearic acid, 12-HpETE, eicosapentaenoic acid methyl ester, 1-linoleoyl glycerol, erucamide, and docosanamide). Betaine, maltose, and sucrose are a group of natural sweeteners, which are widely present in fruits and vegetables and may play important roles in counteracting the sourness of organic acids. The result also showed that the fruit contains a variety of fatty acids, especially polyunsaturated fatty acids.

Considering the disadvantages of LC–MS in identifying highly lipophilic constituents, the hexane extract of *F. tikoua* fruit was analyzed by GC–MS (Figure S4). The result also showed that *F. tikoua* fruit contains high content of different fatty acids. Several other classes of compounds were identified in this fruit (Table S2), including phytosterols (campesterol, stigmasterol, 28-isofucosterol, and sitosterol), triterpenoids (squalene, *α*-amyrin, olean-12-en-3*α*-yl acetate, and urs-12-en-24-oic acid, 3-oxo-, methyl ester), and vitamin E (*δ*-tocopherol and *γ*-tocopherol).

**Figure 2:**
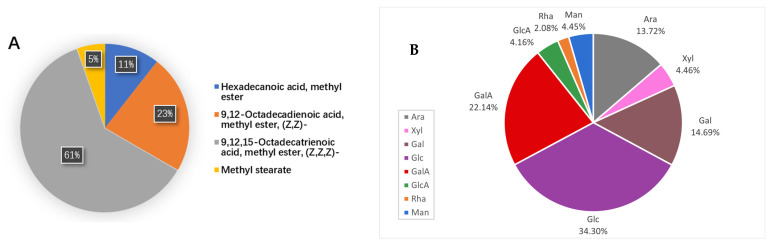
(**A**) Fatty acids composition of *F. tikoua* fruit. (**B**) The ratio of the monosaccharide compositions of the polysaccharide of *F. tikoua* fruit. Each monosaccharide composition is shown in a different color.

In the GC–MS spectrum of the total lipid fraction, the fatty acids were not well separated due to their complexity. Therefore, in order to quantatively analyze the compositions of the fatty acids, which were transformed into fatty acid methyl esters by base-catalyzed methanolysis (KOH in methanol) for further analysis [21], GC–MS analysis showed that the fatty acids in the fruit of *F. tikoua* mainly consisted of hexadecanoic acid (10.56%), 9,12-octadecadienoic acid (22.79%), 9,12,15-octadecatrienoic acid (61.27%), and stearic acid (5.38%) (Figure 2A) and that unsaturated fatty acids accounted for 84.06% of the total fatty acids.

From the above results, it can be concluded that the consumption of a proper amount of *F. tikoua* fruit could provide human some salubrious phytochemicals, such as phytosterols, unsaturated fatty acids, vitamine E, and squalene, which possess beneficial effects on human health [22].

### 2.2. Analysis of Nutritional Phytochemicals

The contents of the crude protein, vitamins, amino acids, total polysaccharides, polyphenols, and flavonoids of *F. tikoua* fruit are reported in Table S3. The *F. tikoua* fruit showed a higher crude protein content (9.41 ± 0.03 g/100 g) compared with other fruits, kiwifruit (0.9 g/100 g) [23], mulberry fruit (1.4 g/100 g) [24], pear (2.6 g/100 g), and prickly pear fruit (0.7 g/100 g) [25], for instance. Figure S5 shows the vitamin composition. It can be seen that *F. tikoua* fruit contains pridoxine, thiamine, niacin, nicotinamide, and ascorbic acid but without riboflavin, cyanocobalamin, or folic acid. Furthermore, it contains a substantial amount of thiamine (205.4 ± 5.4 mg/100 g) and pridoxine (66.6 ± 2.3 mg/100 g). It also showed a high content of total amino acids (9.28%), and aspartic acid (1.48%) and glutamate (1.4%) were at the forefront of all the detected amino acids. In addition, there were also several essential amino acids in this fruit, such as threonine, proline, isoleucine, leucine, phenylalanine, and lysine.

*F. tikoua* fruit possessed a lower total polysaccharide content, with a mean value of 1.25 ± 0.04 g/100 g, than grape, apricot, strawberry, and blueberry (4.89–17 g/100 g) [26]. Thus, it may be used as a potential functional food in the diet of diabetic and obese patients. The analysis of the monosaccharide composition of polysaccharides is of great significance for the study of polysaccharide structure and properties. The monosaccharide composition was shown in Figure S6 and Figure 2B. The monosaccharide composition of this fruit includes glucose (Glc, 34.29%), galacturonic acid (GalA, 22.15%), galactose (Gal, 15.69%), arabinose (Ara, 13.72%), xylose (Xyl, 4.46%), mannose (Man, 4.45%), glucuronic acid (GlcA, 4.17%), and rhamnose (Rha, 2.08%) at an approximate molar ratio of 15:9:7:7:2:2:2:1, among which Glc and GalA are the major monosaccharides.

Moreover, the TFC and TPC were found to be 8.00 ± 0.11 mg/100 g and 0.86 ± 0.01 g/100 g, respectively. In comparison with berries, a reasonably good level of TPC was observed in *F. tikoua* fruit [27]. Phenolic compounds have been shown to reduce oxidative damage in living cells and tissues, suggesting that *F. tikoua* fruit could be good for health [28].

### 2.3. Antioxidant Activity

**Figure 3:**
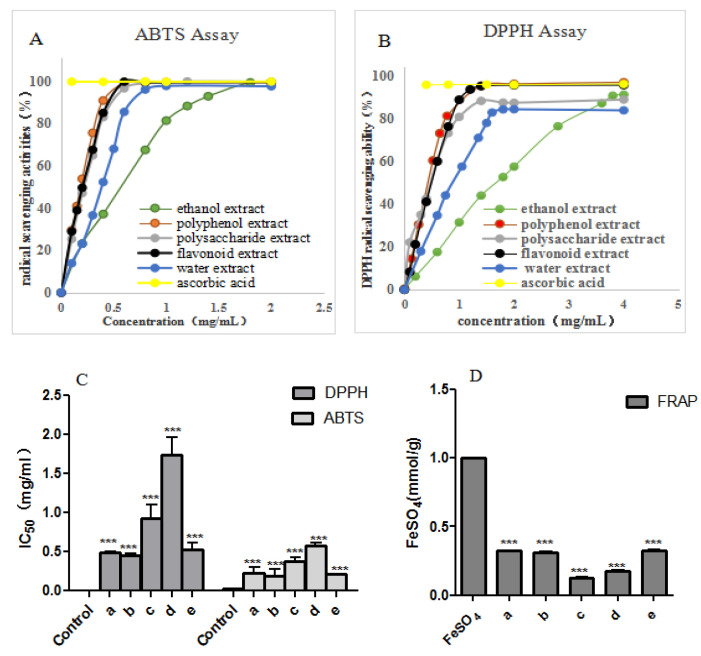
(**A**) ABTS radical scavenging curve of five extracts. (**B**) DPPH radical scavenging curve of five extracts. (**C**) IC_50_ (mg/mL) for ABTS and DPPH free radical scavenging of five extracts. (**D**) FRAP value (mmol/g Fe^2+^) of various extracts of *F. tikoua* fruit. Each bar was replicated three times, with statistical significance defined as *p* < 0.05 (*** *p* < 0.001) vs. control. a = polysaccharide extract; b = polyphenol extract; c = water extract; d = ethanol extract; e = flavonoid extract.

Fruit is one of the main sources of antioxidants in the daily diet, so it is important to estimate its antioxidants and antioxidant capacity. The fruit polyphenols and flavonoids are the major group of natural antioxidants because of their extensive distribution and diversity. Furthermore, a comprehensive analysis with precise information on the antioxidant properties of different extracts including water extracts, alcohol extracts, and polysaccharides is provided, considering the complexity of the phytochemicals. As described in Figure 3A–D, the DPPH and ABTS radical scavenging activities of the five extracts ranged between 0.45–1.74 mg/mL and 0.19–0.58 mg/mL (IC_50_), respectively. In addition, the results of the scavenging ability of five extracts on DPPH and ABTS free radicals were consistent with that of the FRAP method. In DPPH free radical scavenging activity, the extracts of polysaccharide, polyphenols, and flavonoids showed relative higher capability, with IC_50_ values of 0.48 mg/mL, 0.45 mg/mL, and 0.52 mg/mL, respectively. Meanwhile, the polysaccharide extract showed the highest antioxidant activity (0.32 mmol Fe^2+^/g) when measured by FRAP assay. This fruit showed much higher antioxidant activity than that of many daily common fruits such as mango 14.23 (μmol FeSO_4_/g), banana (17.36 μmol FeSO_4_/g), and grapes (0.69 μmol FeSO_4_/g), as reported by Silva [29]. Therefore, the polyphenol, polysaccharide, and flavonoid extracts of this fruit have remarkable antioxidant activity, and it can be inferred that polyphenol and polysaccharide may play key roles in antioxidant ingredients. It is obvious that polyphenols, polysaccharides, and flavonoids are mainly responsible for the antioxidant capacity of this fruit. The water extract may contain highly hydrophilic components such as salts, amino acids, and oligosaccharides. Contrarily, ethanol extract may contain a high content of lipophilic compounds such as fatty acids, sterols, coumarins, and flavones. Neither extracting methods extracted the antioxidant ingredients efficiently, which resulted in their lower antioxidant activity.

### 2.4. Immunomodulatory Activity

**Figure 4:**
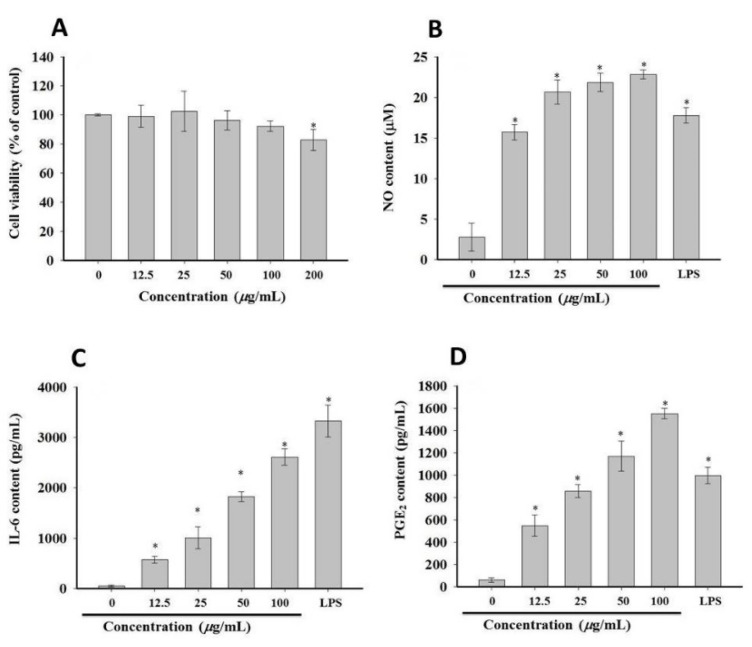
Effect of polysaccharide on the production of inflammatory mediators. (**A**) RAW 264.7 cells treated with different concentrations of polysaccharide, and cell viability was evaluated by MTT assay. (**B**–**D**) The NO content was detected by Griss reagent assay, and IL-6 and PGE_2_ was evaluated by commercial kit. * *p* < 0.05 vs. control group.

As the first line of defense of the body’s immunity, macrophages are considered to be important target cells for polysaccharides, which in turn promote the secretion of downstream effector molecules, such as NO, PGE_2_, IL-6, IL-1β, and TNF-α [30]. The release of these cytokines also reflects the effects of polysaccharides on the immune response to exogenous substances. As shown in Figure 4A, compared with the control group, *F. tikoua* polysaccharides had no obvious effect on the growth of RAW 264.7 cells (*p* > 0.05), with the maximum relative viability being 102.53% of control at 25 μg/mL. Furthermore, the result of NO production showed that, as the concentration of polysaccharide increased, the production of NO also increased. In addition, when the concentration of the polysaccharide was increased to 5.0 μg/mL, the production of NO was significantly higher than that of the LPS group (Figure 4B). The promotion of NO production indicates that the polysaccharides of *F. tikoua* could activate the bactericidal and tumoricidal activity of macrophages and also may be a potential immunostimulant.

As a kind of protein secreted by immune cells, IL-6 is associated with phagocytosis, antigen presentation, and inflammatory regulation. The effect of polysaccharides on IL-6 secretion in RAW 264.7 cells was evaluated by ELISA, and the results are shown in Figure 4C,D, in which the level of stimulated IL-6 gradually increased as the concentration of polysaccharide increased. Although the level of IL-6 secreted by polysaccharide-treated RAW 264.7 cells was lower than that of LPS-treated cells, the results indicate that polysaccharides activate the immune system by stimulating macrophages to secrete IL-6.

**Figure 5:**
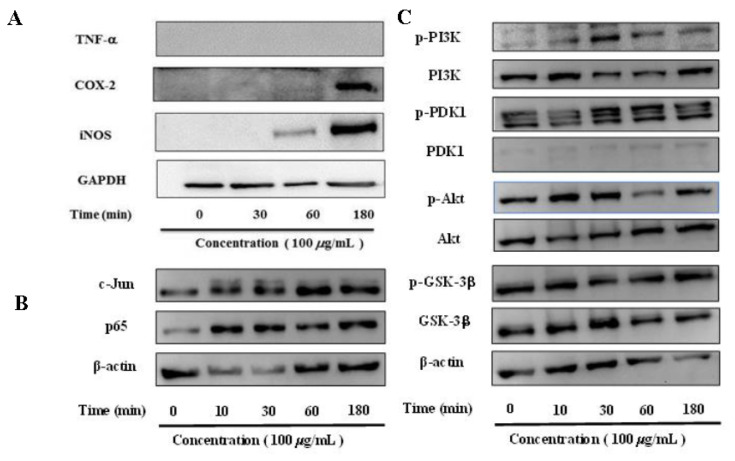
(**A**) The effect of polysaccharide on the protein expression of iNOS, COX-2, and TNF. (**B**) The effect of polysaccharide on the translocation of transcription factors in RAW264.7 cells. (**C**) The effect of polysaccharide on the PI3K/AKT signaling pathway in RAW264.7 cells.

As shown in Figure 5A, the absence of a TNF-*α* band indicates that the polysaccharide has no significant effect on the expression of the gene. However, the expression of iNOS and COX-2 continued to increase with the prolongation of the stimulation time of the polysaccharide. The result indicated that polysaccharide can promote the expression of iNOS and COX-2, thereby enhancing the immune function of macrophages.

### 2.5. Effect of Polysaccharide on NF-κB Translocation in RAW 264.7 Cells

NF-κB is a ubiquitous transcription factor, and the transcription subunit p65 is a key factor involved in the activation of pro-inflammatory cytokines (e.g., iNOS, IL-6, and TNF-α) [31]. Once activated, NF-κB is translocated to the nucleus and modulates the expression of target genes [30]. As shown in Figure 5B, polysaccharide stimulation rapidly enhanced the levels of c-Jun and p65 within 10 min and reached its maximum level at 30 min, 1 h, and 3 h, respectively. Therefore, polysaccharide significantly decreased the expression of c-Jun and p65 proteins in the cytoplasm and increased the translocation of c-Jun and p65 to the nucleus.

### 2.6. Effects of Polysaccharide on PI3K/AKT Signaling Pathway in RAW 264.7 Cells

PI3K/Akt are members of the signaling pathway that plays an important role in modulating the immune response and NF-κB signal transduction [32]. The results suggested that significant phosphorylation of Akt, PI3K, and PDK1 occurred from 10 to 180 min after polysaccharide treatment (Figure 5C), which suggested that the ploysaccharide of *F. tikoua* fruit exerted immuno-modulation via the PI3K/AKT signaling pathway.

## 3. Experimental

### 3.1. Materials and Chemicals

3,4,5-Dimethylthiazol-2-yl-2,5-diphenyltetrazolium bromide (MTT) was purchased from Biofroxx (Einhausen, Hessen, Germany). Bovine serum albumin (BSA) was bought from VWR Life Science (Amresco, Solon, OH, USA). RPMI medium 1640 and a penicillin–streptomycin solution were purchased from Gibco BRL (Thermo Fisher Scientific, Shanghai, China). Lipopolysaccharide (LPS), sulfanilamide, and *N*-1-napthylethylenediamine dihydrochloride were bought from Sigma-Aldrich (St. Louis, MO, USA). Fetal bovine serum (FBS) was obtained from Corning (Medford, MA, USA). Primary antibodies including iNOS and COX-2 were acquired from Cell Signaling Technology (Beverly, MA, USA). Secondary antibodies including goat anti-rabbit IgG H&L (ab6721) were purchased from Abcam (Cambridge, MA, USA). The enhanced chemiluminescence (ECL) Western blot kit, RIPA lysis buffer, and BCA protein assay kit were obtained from CWBIO (Taizhou, China). The NMR spectra were acquired on a Bruker Avance III 400 MHz NMR spectrometer. Open column chromatography was performed on silica gel (200–300 mesh, Qingdao Marine Chemistry Co., Ltd., Qingdao, China) and Sephadex LH-20 (40–70 μm, Amersham Pharmacia Biotech AB, Uppsala, Sweden). Other solvents are all chromatographic or analytical grades and purchased from Kelon Reagent Co., Ltd. (Chengdu, China).

The ripe fruit of *F. tikoua* was collected from Beichuan County, Sichuan Province, China, and authenticated by associate professor Yan Ren, Southwest Minzu Univisity. A voucher specimen (LMFT2002) has been deposited in the College of Pharmacy, Southwest Minzu University.

### 3.2. Isolation and Quantification of Main Constituents

The air-dried powdered fruit of *F. tikoua* (200.9 g, 50 mesh) were extracted with methanol (1.5 L) at 60 °C three times (each 1 h). The extract (45.0 g, 22.4%) was obtained after concentration under vacuum. Then the extract was subjected to silica gel elution successively with a petroleum ether/ethyl acetate gradient (100:1→1:1) and a chloroform/methanol gradient (5:1→1:1) to give eight fractions (A–H). Fraction B (371.9 mg) was subjected to a silica gel column eluted by petroleum ether/ethyl acetate gradient (10:1→1:1) to afford a mixture of **1** (β-sitosterol) and **2** (stigmasterol) (60.2 mg), **3** (psoralen, 104.9 mg), and **4** (5-methoxypsoralen). Fraction E was purified by Sephadex LH-20 (chloroform/methanol, 1:1) to yield 5 fractions (EA1–5). Fraction EA2 was subjected to silica gel elution (chloroform/methanol, 8:1) to yield **5** (protocatechuic acid methyl ester, 55.7 mg). Fraction F (507.9 mg) was purified by a silica gel column (chloroform/methanol, 10:1) to afford **6** (daucosterin, 85.0 mg).

Then the content of psoralen (**3**) was analyzed by the HPLC method [33]. The HPLC conditions were as follows: column, Waters (250 × 4.6 mm, 5 μm); mobile phase, acetonitrile/water (40:60, *v*/*v*); flow rate, 1.0 mL/min; column temperature, 35 °C; detector, 247 nm. The content of protocatechuic acid methyl ester (**5**) was also determined by HPLC with the mobile condition of acetonitrile/water (15:85, *v*/*v*).

### 3.3. LC–MS and GC–MS Analyses of the Chemical Components in F. tikoua Fruit

LC–MS analyses were performed on a Thermo Scientific Q Exactive equipped with an Accucore 2.6 μm C18 column (100 × 2.1 mm). Analysis conditions were as follows: flow rate 0.3 mL/min, injection volume 0.5 μL, and column oven temperature 35 °C, and the mobile phase consisted of methanol (A) and water (B). The applied gradients, with a flow rate of 0.3 mL/min, were as follows: 5–95% A for 0–25.0 min and 95% of mobile phase A for 25.0–30.0 min. GC–MS analyses were carried out on a 7890A/5975C instrument equipped with a HP-5MS column (30 m × 0.25 mm, 0.25 μm, Agilent, Santa Clara, CA, USA) in full-scan mode (*m*/*z* 40–700). GC conditions were (a) inlet temperature, 210 °C; (b) injection volume, 5 μL; (c) inlet mode, split (4:1); (d) column flow rates, 1.0 mL/min; (e) transfer line, 250 °C; (f) oven program, 90 °C for 3.0 min; and then 10 °C/min to 160 °C, held for 5.0 min; then 3 °C/min to 280 °C, held for 40 min; and then 280 °C, held for 17 min, for a 65.0 min total run time.

### 3.4. Chemical Analysis

#### 3.4.1. Proximate Analysis

The crude protein of *F. tikoua* fruit was determined by the Kjeldahl method, and the content was calculated by multiplying the percentage of nitrogen in the digestion by 6.25. Data were expressed as mass percent in 100 g of dried sample (%). The ash content was measured by a Muffle furnace at 550 °C for 4 h and calculated as g/100 g of the dried sample [34].

#### 3.4.2. Vitamin Analysis

The content of water-soluble vitamins including pyridoxine, thiamine, riboflavin, ascorbic acid, cyanocobalamin, niacin, folic acid, and nicotinamide was determined by HPLC with a UV detector at wavelengths of 210 nm and 254 nm and a C18 column (Waters, 250 × 4.6 mm, 5 μm) [35]. The conditions were as follows: injection volume 10 μL and column oven temperature 30 °C; the mobile phase consisted of methanol (A) and a KH_2_PO_4_ solution, pH = 4.0 (B). The flow rate was held constant at 1.0 mL/min, with a stepwise gradient of 5%, 5%, 10%, and 40% of solvent A at 0, 5, 15, and 35 min, respectively. The result was expressed as a standard equivalent of the dried sample (mg/100 g).

#### 3.4.3. Amino Acid Composition

Amino acid determination was performed according to the GB method [36]. Results were expressed as the mass percentage of the dried sample (%).

#### 3.4.4. Total Polysaccharide Content and Its Monosaccharide Composition

The polysaccharide was isolated from the fruit of *F. tikoua* by hot water extraction and ethanol precipitation [37]. In brief, the sample (1.0 g) was extracted with hot water (49.0 mL) at 90 °C for twice (each 21 min). Then the filtrate was concentrated with a rotary evaporator and precipitated with ethanol. The precipitate was successively washed with ethanol, acetone, and petroleum ether and dried to give a crude polysaccharide after centrifugation. The polysaccharide content was determined using the sulfuric-acid–phenol method. Result was expressed as glucose equivalent per gram of dry sample (g GE/100 g) [38].

The analysis was determined by the PMP pre-column derivative method [39]. Briefly, a polysaccharide aqueous solution (10 mg/mL) was first hydrolyzed by 2 M TFA at 105 °C for 4 h, followed by neutralization with a 2 M NaOH aqueous solution. Then the standard monosaccharide and the hydrolysate were respectively mixed with a ribose internal standard solution and successively converted into its PMP derivatives. The aqueous layer was filtered through a 0.22 μm membrane for HPLC analysis. The HPLC was equipped with a PDA detector and a YMC-Pack ODS-AQ column (4.6 × 250 mm, 5 µm). The mobile phase was a binary gradient elution of a triethylamine–ammonium acetate aqueous solution (A) and acetonitrile (B) with 1.0 mL/min. The results were expressed as the molar ratio of each monosaccharide.

#### 3.4.5. Total Flavonoid Content (TFC)

Total flavonoid content was determined according to the following method with some modification [40]. Briefly, the dried sample (5.0 g) was ultrasonically extracted with 50 mL 80% ethanol at 45 °C for 30 min and filtered. This procedure was repeated three times. The filtrate was combined to obtain the ethanol extract. A total of 1 mL of the ethanol extract was placed in a 10 mL volumetric flask, in which distilled water was added to 5 mL, and then 0.3 mL of NaNO_2_ was added. After 5.0 min, a total of 0.3 mL of AlCl_3_ was added and kept for another 6 min. Then 2 mL of 1 M NaOH was added, and the total volume was made up to 10 mL with distilled water. The solution was kept for 30 min. Absorbance was measured against a blank at 510 nm (Perkin elmer lambda 35 UV/Vis spectrum, Waltham, MA, USA), and the flavonoid content was determined as the rutin equivalent from a calibration curve of rutin standard solutions and expressed as milligrams of rutin/100 g of dried sample. All measurements were performed in triplicate.

#### 3.4.6. Total Polyphenols Content (TPC)

According to the method of Cai [40], the dried sample (1.0 g) was extracted with 20 mL 45% MeOH containing 0.1% HCl in a water bath at 60 °C to obtain the extract of TPC. A total of 1 mL of the sample solution was mixed with 1 mL of Folin–Ciocalteu reagent. After 1 min of incubation at room temperature, 1.5 mL of a 20% Na_2_CO_3_ aqueous solution was added to the mixture, followed by the addition of 7.5 mL of distilled water; the solution was then kept in a constant-temperature water bath at 70 °C for 10 min. After cooling to room temperature, absorbance was measured at 765 nm. The TPC was expressed as a gallic acid equivalent (GAE) from the calibration curve of gallic acid standard solutions and expressed as milligrams of GAE/100 g. All samples were performed in triplicate.

### 3.5. Antioxidant Activity Analysis

#### 3.5.1. DPPH Assay

The polysaccharide, polyphenol, water, ethanol, and flavonoid extracts were obtained from the fruit of *F. tikoua*. More precisely, the extract of polysaccharide was obtained by the method for total polysaccharide content (Section 3.4.4). Polyphenol and flavonoid extracts were prepared as described above for the preparation of TPC (Section 3.4.6) and TFC (Section 3.4.5). While water and ethanol extracts were extracted with water (60 °C, 3 × 1 h) and ethanol (45 °C, 3 × 1 h), respectively. The DPPH radical scavenging activity of these extracts was determined based on the method of Li [41]. Each extract (2.0 mL) was mixed with a DPPH solution (2.0 mL, 2.0 × 10^−4^ M) and kept at room temperature for 30 min in the dark. Absorbance was measured at 515 nm, and ascorbic acid was used as a standard control. Then the IC_50_ values were calculated to represent the results (mg/mL).

#### 3.5.2. FRAP Assay

The FRAP assay was determined referring to the method of Sánchez-González [37]. Fresh FRAP reagent (TPTZ, FeCl_3_, and potassium acetate buffer were mixed at a ratio of 1:1:10 and then incubated at 37 °C for 15 min, 3.0 mL) was mixed with 1.0 mL of diluted samples, and absorbance was recorded at 595 nm after reacting at 37 °C for 30 min. The total antioxidant capacity (FRAP) was estimated from a standard curve of ferrous sulfate standard solutions. Results were expressed as the amount of substance equivalent to Fe^2+^ per gram of extract (mmol Fe^2+^/g).

#### 3.5.3. ABTS Assay

ABTS radical scavenging activity was determined according to the method described by Apea-Bah with slight modifications [42]. The ABTS stock solution was diluted with ethanol to obtain an absorbance of about 0.70 at 734 nm. The appropriately diluted extracts (2.0 mL) were added to fresh ABTS radical solution (2.0 mL). The absorbance at 734 nm was read after 10 min of reaction. With ascorbic acid as a positive control, the free radical scavenging rate and IC_50_ values (mg/mL) were calculated in the same way as the FRAP assay.

### 3.6. Immunomodulatory Activity Analysis

#### 3.6.1. Cell Line and Cell Culture

RAW 264.7 cells were obtained from the American Type Culture Collection (Rockville, MD, USA) and maintained in RPMI 1640 medium with 10% FBS, 100 μg/mL streptomycin, and penicillin (100 U/mL) at 37 °C with 5% CO_2_.

#### 3.6.2. Cell Viability Assay

Briefly, RAW 264.7 cells were suspended at a density of 1 × 10^5^ cells/well. After 24 h incubation, a series of concentrations of the polysaccharide solution were added and incubated at 37 °C for an additional 24 h. The cytotoxicity of polysaccharide on RAW 264.7 cells was assessed by the MTT method [43].

#### 3.6.3. NO, PGE2, and IL-6 Production

RAW 264.7 cells (1 × 10^5^ cells/well) were cultured for 18 h in a 96-well plate. Cells were then stimulated with various concentrations of polysaccharide for 24 h. The nitric oxide level was determined through the Griess reagent as described previously [44]. The levels of PGE2 and IL-6 in the culture supernatants were determined using an ELISA kit (Abcam).

#### 3.6.4. Western Blot Analysis

RAW 264.7 cells were seeded at 5 × 10^6^ cells/well onto 60 mm plates for 18 h. After being treated with a polysaccharide solution for different time points, the cells were collected, and protein concentrations were determined by a BCA protein assay kit (Absin, Shanghai, China). The protein samples were boiled at 95 °C for 5 min and separated using a 10% SDS-polyacrylamide gel for 2 h, then transferred onto PVDF membranes. After being blocked with 5% BSA for 2 h at room temperature, the membrane was incubated overnight with primary antibodies at 4 °C and for 2 h with secondary antibodies conjugated horseradish peroxidase (HRP) at room temperature used as 1:2000 dilutions. The protein bands were visualized with an eECL Western Blot kit (CWBio, Beijing, China) and photographed using the Tanon-5200 system (Tanon 5200 Multi, Beijing, China).

### 3.7. Statistical Analysis

The analysis was performed in triplicate, and results were expressed as the mean of three independent experiments (*n* = 3). Statistical analysis of the data was performed using GraphPad Prism 5 and IBM SPSS statistics software(19.0), and multigroup results were compared using one-way ANOVA (and norparametic). *p* values of less than 0.05 were considered significant.

## 4. Conclusions

This study aimed to provide some nutraceutical features of *F. tikoua* fruit, which could help to enhance its utilization as a source of functional food material. The fruit has a considerable protein and amino acid content and contains essential amino acids such as threonine, valine, isoleucine, leucine, and so on. To some extent, it can be proved that the fruit of *F. tikoua* has certain nutritional value. As shown here, this underutilized fruit has a high polyphenol content and ideal FRAP and DPPH values, which could be an excellent source of antioxidants. Moreover, the polysaccharide has the function of improving immunity, which indicates that it may be beneficial for treating diseases, and can be used as a potential natural immunomodulator in the field of functional food.

## Data Availability

Not applicable.

## Supplementary Materials

The following supporting information can be downloaded at https://www.mdpi.com/article/10.3390/molecules27206880/s1 , Figure S1: The picture of *Ficus tikoua* fruit; Figure S2: TIC LC-MS spectrum of the methanol extract of *F. tikoua* fruit; Figure S3: HPLC spectrum of the methanol extract of *F. tikoua* fruit; Figure S4: TIC GC-MS spectrum of the hexane extract of *F. tikoua* fruit; Figure S5: The HPLC analysis result of the vitamin composition of *F. tikoua* fruit; Figure S6: The result of the monosaccharide composition of *F. tikoua* fruit; Table S1: Compounds identified by LC-MS from the methanol extract of *F. tikoua* fruit; Table S2: Compounds identified by GC-MS from the hexane extract of *F. tikoua* fruit.; Table S3: Crude protein, amino acids, total polysaccharides, polyphenols, flavonoids, and vitamins contents of *F. tikoua* fruit.

## Sample Availability

Samples of the compounds **1**–**6** are available from the authors.
