# Supplementary material for: Systematical Ingredient Investigations of Ficus tikoua Bur. Fruit and Immunoregulatory and Antioxidant Effects of Different Fractions
Source: Molecules. 2022 Oct 14;27(20):6880. doi: 10.3390/molecules27206880 (PMC9608957; doi:10.3390/molecules27206880)

Systematical Ingredient Investigations of *Ficus tikoua* Bur. Fruit and Immunoregulatory and Antioxidant Effects of Different Fractions

Yu Gong[1,2], Wei Luo[2], Hulan Chen[2], Bo Ren[2], Weicheng Hu[3*], Limei Li[1*]

[1] College of Pharmacy, Southwest Minzu University, Chengdu 610041, China.

[2] Key Laboratory of Standardization of Chinese Herbal Medicine, Ministry of Education, State Key Laboratory of Southwestern Chinese Medicine Resources, Chengdu University of Traditional Chinese Medicine, Chengdu 611137, China.

[3] Jiangsu Collaborative Innovation Center of Regional Modern Agriculture & Environmental Protection, Jiangsu Key Laboratory for Eco-Agricultural Biotechnology around Hongze Lake, Huaiyin Normal University, Huaian 223300, China.

[*]Correspondence: hu_weicheng@163.com (W.-C. H.); limeili@swun.edu.cn (L.-M. L.)

CONTENTS

Abbreviations...3

Figure S1. The picture of *Ficus tikoua* fruit 5

Figure S2. TIC LC-MS spectrum of the methanol extract of *F. tikoua* fruit.....................................6

Figure S3. HPLC spectrum of the methanol extract of *F. tikoua* fruit..6

Table S1. Compounds identified by LC-MS from the methanol extract of *F. tikoua* fruit................7

Figure S4. TIC GC-MS spectrum of the hexane extract of *F. tikoua* fruit..9

Table S2. Compounds identified by GC-MS from the hexane extract of *F. tikoua* fruit.................10

Table S3. Crude protein, amino acids, total polysaccharides, polyphenols, flavonoids, and vitamins contents of *F. tikoua* fruit...12

Figure S5. The HPLC analysis result of the vitamin composition of *F. tikoua* fruit 14

Figure S6. The result of the monosaccharide composition of *F. tikoua* fruit 15

Abbreviations

ABTS 2,2'-azino-bis(3-ethylbenzothiazoline-6-sulfonic acid)

Ara arabinose

BCA bicinchoninic acid

BSA bovine serum albumin

DPPH 2,2-diphenyl-1-picrylhydrazyl radical

ECL enhanced chemiluminescence

ELISA enzyme linked immunosorbent assay

FBS fetal bovine serum

FRAP ferric reducing antioxidant power

Gal galactose

GalA galacturonic acid

GC-MS gas chromatography-mass spectrometry

Glc glucose

GlcA glucuronic acid

HPLC high performance liquid chromatography

HRP horseradish peroxidase

IC_{50} half maximal inhibitory concentration

LC-MS liquid chromatography-mass spectrometry

LPS lipopolysaccharide

Man mannose

MTT 3,4,5-dimethylthiazol-2-yl-2,5-diphenyltetrazolium bromide

NMR	nuclear magnetic resonance
NO	nitric oxide
PGE_2	prostaglandin E2
PMP	1-phenyl-3-methyl-5-pyrazolone
PVDF	poly vinylidene fluoride
Rha	rhamnose
SDS	sodium dodecyl sulfate
TFA	trifluoroacetic acid
TFC	total flavonoids content
TPC	total polyphenols content
TPTZ	tri-2-pyridyl-s-triazine
UV	ultraviolet
Xyl	xylose

Figure S1. The picture of *Ficus tikoua* fruit.

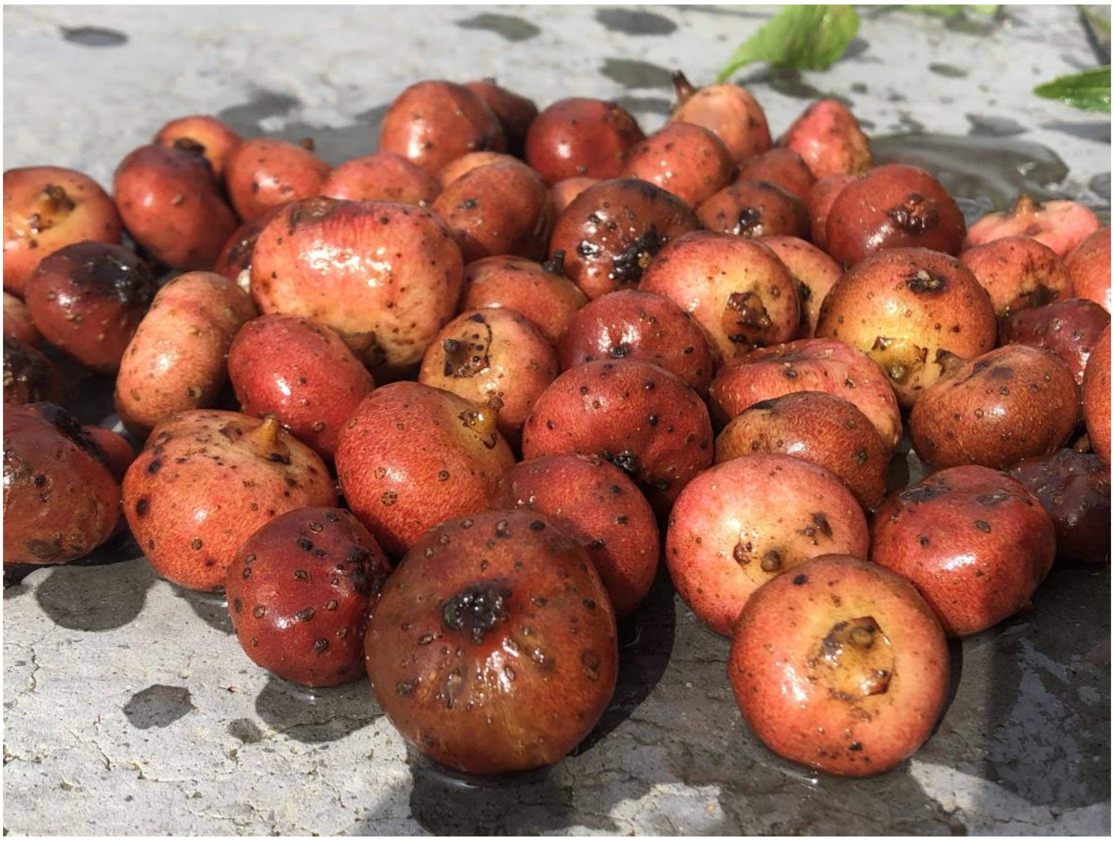

Figure S2. TIC LC-MS spectrum of the methanol extract of *F. tikoua* fruit.

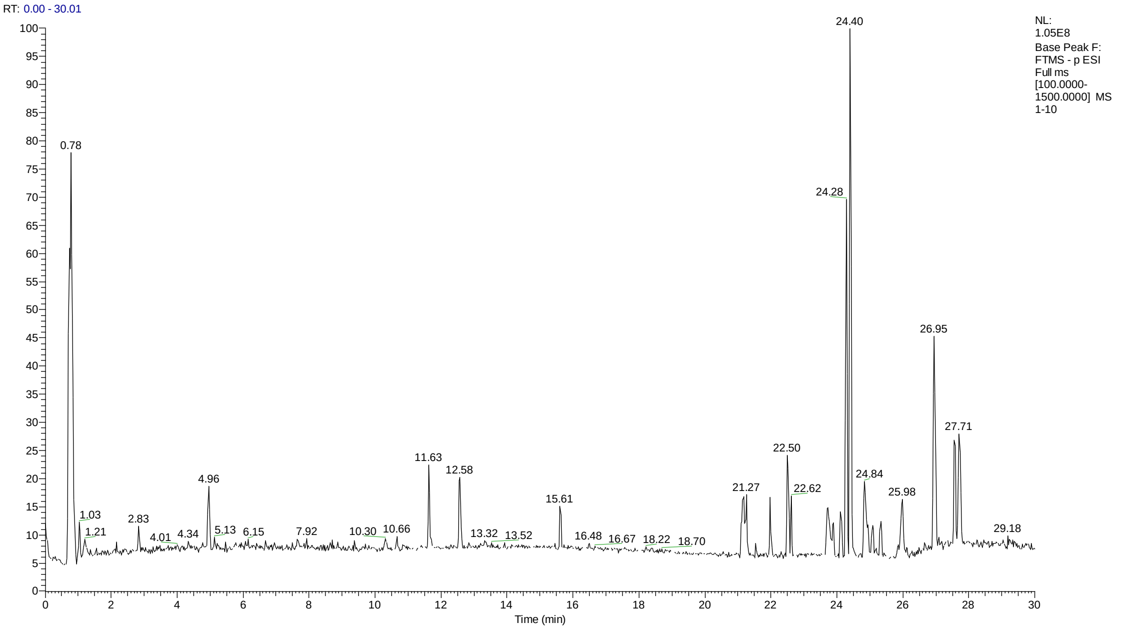

Figure S3. HPLC spectrum of the methanol extract of *F. tikoua* fruit.

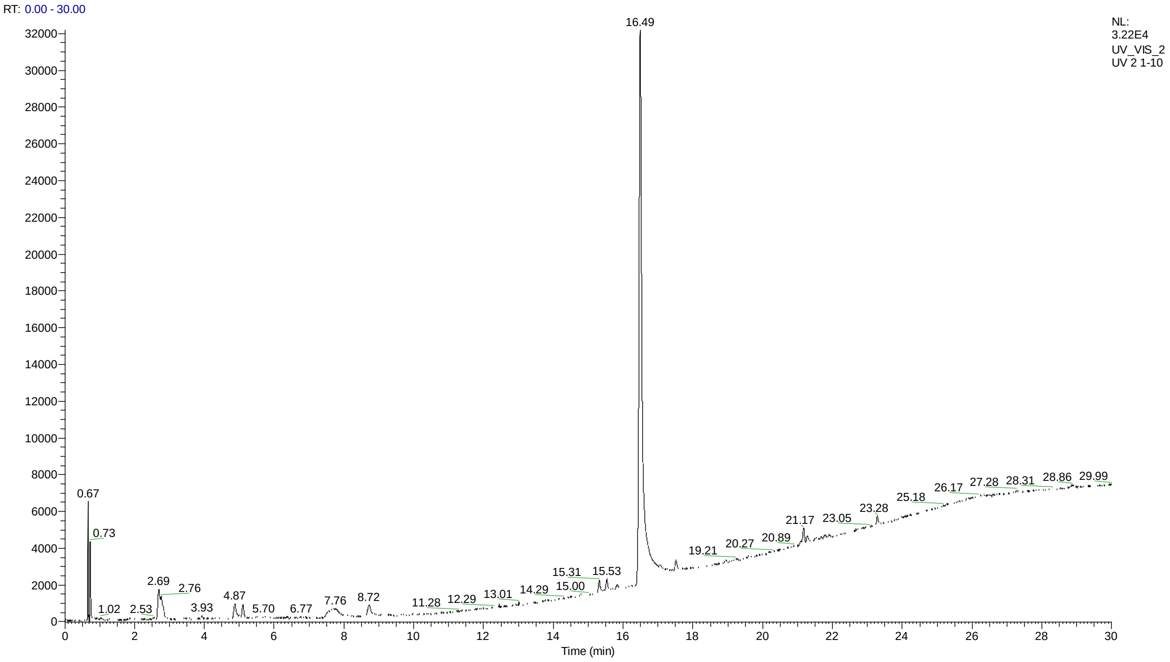

Table S1. Compounds identified by LC-MS from the methanol extract of *F. tikoua* fruit.

No.	t_R (min)	compound name	molecular weight	molecular formula	structure
1	0.7	choline	103.1	$C_5H_{13}NO$	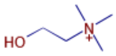
2	0.7	asparagine	132.1	$C_4H_8N_2O_3$	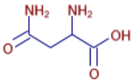
3	0.73	betaine	117.1	$C_5H_{11}NO_2$	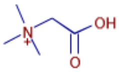
4	0.74	*D*-(+)-proline	133.1	$C_5H_9NO_2$	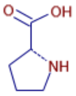
5	0.74	*D*-(+)-maltose	364.1	$C_{12}H_{22}O_{11}$	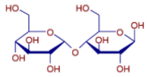
6	0.74	sucrose	342.1	$C_{11}H_{22}O_{11}$	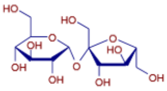
7	0.74	*D*-(-)-quinic acid	192.1	$C_7H_{12}O_6$	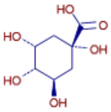
8	0.77	pipecolic acid	129.1	$C_6H_{11}NO_2$	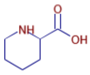
9	0.79	*DL*-malic acid	134.0	$C_4H_6O_5$	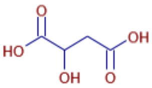
10	1.03	citric acid	192.0	$C_6H_8O_7$	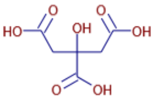
11	1.19	methylmalonic acid	118.0	$C_4H_6O_4$	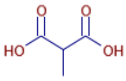
12	1.23	*DL*-norleucine	131.1	$C_6H_{13}NO_2$	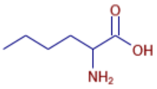
13	2.06	*L*-phenylalanine	165.1	$C_9H_{11}NO_2$	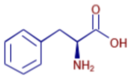
14	2.84	8-hydroxyquinoline	145.1	C_9H_7NO	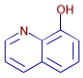
15	2.84	4-indolecarbaldehyde	145.1	C_9H_7NO	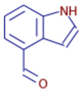

16	3.46	indole-3-acrylic acid	187.1	$C_{11}H_9NO_2$	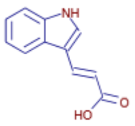
17	5.57	neochlorogenic acid	354.1	$C_{16}H_{18}O_9$	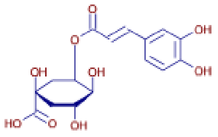
18	6.44	chlorogenic acid	354.1	$C_{16}H_{18}O_9$	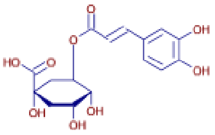
19	11.44	psoralen	186.0	$C_{11}H_6O_3$	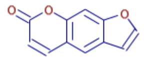
20	19.44	8-prenylnaringenin	340.1	$C_{20}H_{20}O_5$	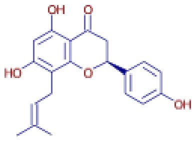
21	21.22	9*S*,13*R*-12-oxophytodienoic acid	292.2	$C_{18}H_{28}O_3$	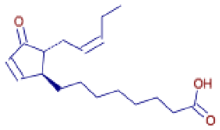
22	21.82	9-oxo-10(*E*),12(*E*)-octadecadienoic acid	294.2	$C_{18}H_{30}O_3$	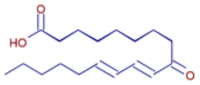
23	21.99	*α*-eleostearic acid	278.2	$C_{18}H_{30}O_2$	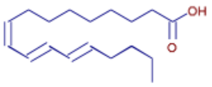
24	22.00	(+/-)12-HpETE	318.2	$C_{20}H_{32}O_4$	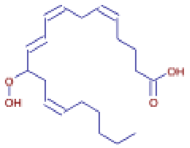
25	23.43	eicosapentaenoic acid methyl ester	322.2	$C_{21}H_{32}O_2$	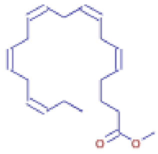
26	24.46	1-linoleoyl glycerol	354.3	$C_{21}H_{38}O_4$	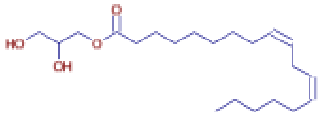
27	26.96	erucamide	337.3	$C_{22}H_{43}NO$	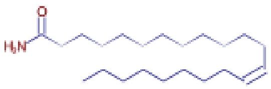
28	28.44	docosanamide	339.3	$C_{22}H_{45}NO$	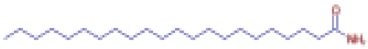

Figure S4. TIC GC-MS spectrum of the hexane extract of *F. tikoua* fruit.

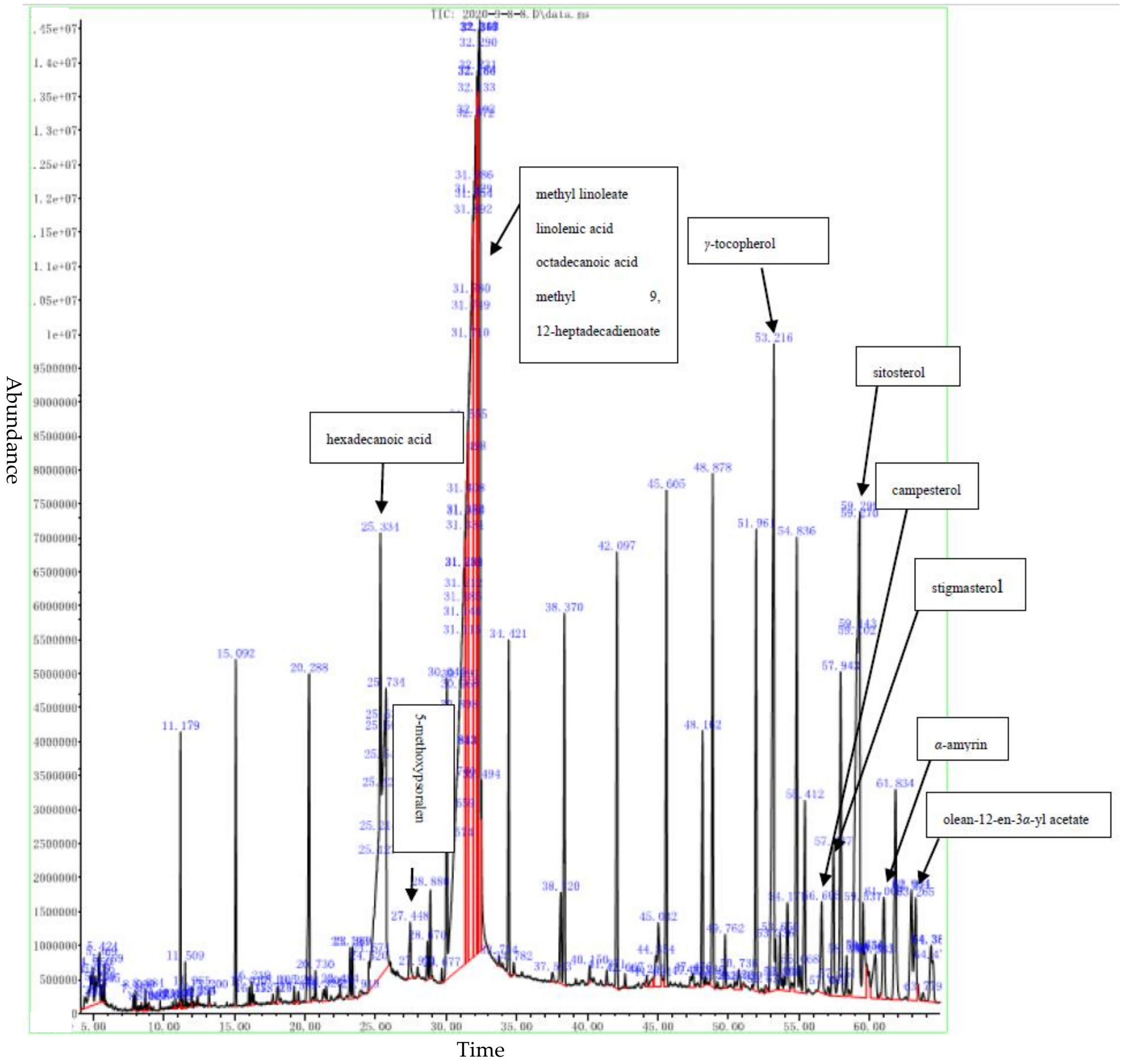

Table S2. Compounds identified by GC-MS from the hexane extract of *F. tikoua* fruit.

t_R (min)	compound name	CAS No.	relative area %
10.827	2,6-di-tert-butylbenzoquinon	719-22-2	0.02
11.507	2,4-di-tert-butylphenol	96-76-4	0.1
13.198	*n*-hexadecane	544-76-3	0.03
16.017	*n*-heptadecane	629-78-7	0.05
18.106	tetradecanoic acid	544-63-8	0.09
19.227	octadecane	593-45-3	0.05
19.540	6,10,14-trimethyl-2-pentadecanone	502-69-2	0.1
21.323	pentadecanoic acid	1002-84-2	0.07
22.487	nonadecane	629-92-5	0.03
23.192	7,9-ditert-butyl-1-oxaspiro[4.5]deca-6,9-diene-2,8-dione	82304-66-3	0.18
23.370	hexadecanoic acid, methyl ester	112-39-0	0.16
23.921	16-methyl-oxacyclohexadecan-2-one	4459-57-8	0.04
25.128	hexadecanoic acid	57-10-3	1.52
27.450	5-methoxypsoralen	484-20-8	0.3
27.971	1-nonadecene	18435-45-5	0.03
28.670	methyl linoleate	112-63-0	0.13
28.878	linolenic acid, methyl ester	301-00-8	0.33
29.675	methyl stearate	112-61-8	0.05
30.661	linoelaidic acid	506-21-8	0.57
30.845	linolenic acid	463-40-1	3.09
30.900	linolenyl alcohol	506-44-5	0.61
30.967	linoleic acid	60-33-3	0.84
31.378	methyl 9, 12-heptadecadienoate	29305-60-0	0.38
32.493	octadecanoic acid	57-11-4	0.74
33.737	eicosane	112-95-8	0.04
34.785	phytane	638-36-8	0.04
37.542	tetracosane	646-31-1	0.06
40.152	pentacosane	629-99-2	0.06
44.852	2-linoleoyl glycerol	3443-82-1	0.21
48.161	squalene	111-02-4	1.01
50.734	δ-tocopherol	119-13-1	0.14
53.216	γ-tocopherol	7616-22-0	4.52
53.651	1-docosene	1599-67-3	0.22
53.804	nonacos-1-ene	18835-35-3	0.04
56.604	campesterol	474-62-4	0.65
57.437	stigmasterol	83-48-7	0.95
59.104	sitosterol	83-46-5	5.36
59.539	28-isofucosterol	481-14-1	0.48
61.010	α-amyrin	638-95-9	0.74

62.952	olean-12-en-3*α*-yl acetate	33055-28-6	0.58
64.337	urs-12-en-24-oic acid, 3-oxo-, methyl ester	20475-86-9	0.26

Table S3. Crude protein, amino acids, total polysaccharides, polyphenols, flavonoids, and vitamins contents of *F. tikoua* fruit.

phytochemical	standard curve line	result
crude Protein	-	9.41 ± 0.03 (g/100g)
ash	-	10.29 ± 0.08 (g/100g)
vitamin	-	-
pridoxine	-	66.67 ± 2.3 (mg/100g)
thiamine	-	205.42 ± 5.4 (mg/100g)
riboflavin	-	0
ascorbic acid	-	10.00 ± 0 (mg/100g)
cyanocobalamin	-	0
niacin	-	3.20 ± 0.4 (mg/100g)
folic acid	-	0
nicotinamide	-	15.40 ± 1.3 (mg/100g)
amino acids	-	9.28%
threonine	-	0.35%
proline	-	0.51%
isoleucine	-	0.40%
leucine	-	0.69%
phenylalanine	-	0.48%
lysine	-	0.51%
aspartic acid	-	1.48%
serine	-	0.50%

glutamate	-	1.40%
alanine	-	0.49%
glycine	-	0.44%
cystine	-	0.21%
methionine	-	0.12%
tyrosine	-	0.24%
histidine	-	0.35%
arginine	-	0.62%
proline	-	0.47%
total polysaccharide	Y = 17.902x + 0.05, R^2 =	1.25 ± 0.04 (g/100g)
total polyphenols (TPC)	0.9970	0.86 ± 0.01 (g/100g)
total flavonoids (TFC)	Y = 28.611x - 0.424, R^2 =	8.00 ± 0.11 (mg/100g)
	0.9951	
	Y = 3.4967x - 0.0467, R^2 =	
	0.9969	

Each value was expressed as mean ± SE (*n* = 3).

Figure S5. The HPLC analysis result of the vitamin composition of *F. tikoua* fruit.

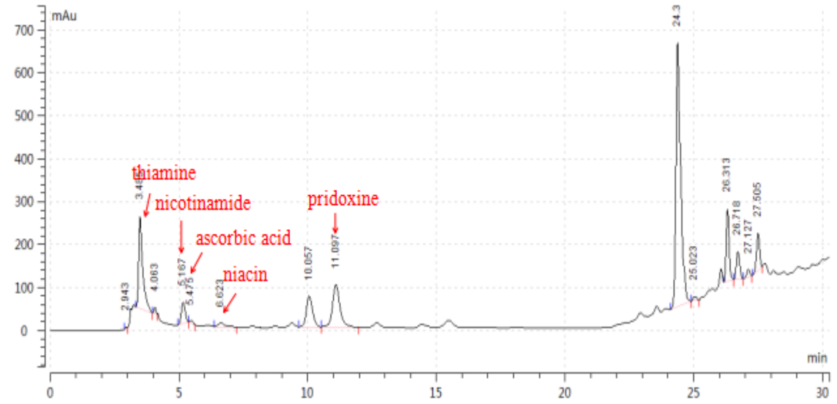

Figure S6. The result of the monosaccharide composition of *F. tikoua* fruit. (A) The result of the HPLC spectrum of the PMP-labeled monosaccharide standard. (B) The result of the monosaccharide composition of *F. tikoua* fruit.

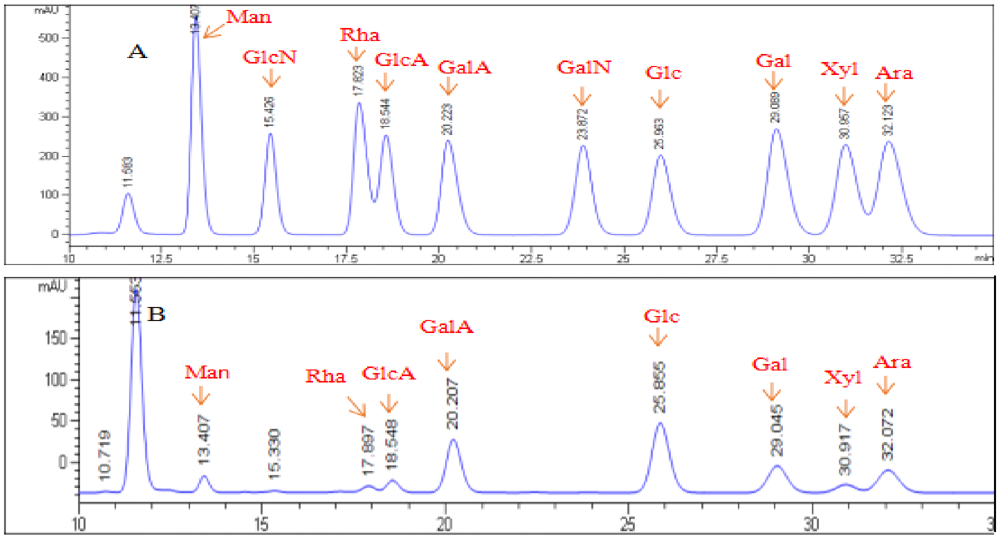

Supplement: Supplementary file 1 [file molecules-27-06880-s001.zip › molecules-1946241-supplementary .pdf]
